# A Prospective Comparison of EUS-Guided FNA Using 25-Gauge and 22-Gauge Needles

**DOI:** 10.1155/2009/546390

**Published:** 2009-11-17

**Authors:** Hiroo Imazu, Yujiro Uchiyama, Hiroshi Kakutani, kei-ichi Ikeda, Kazuki Sumiyama, Mitsuru Kaise, Salem Omar, Tiing Leong Ang, Hisao Tajiri

**Affiliations:** ^1^Department of Endoscopy, The Jikei University School of Medicine, Tokyo 105-8461, Japan; ^2^Division of Gastroenterology, Department of Medicine, Faculty of Medicine, University of Malaya, Kuala Lumpur 50603, Malaysia; ^3^Division of Gastroenterology, Department of Medicine, Changi General Hospital, Singapore 529889; ^4^Division of Gastroenterology and Hepatology, Department of Internal Medicine, The Jikei University, Tokyo 105-8461, Japan

## Abstract

*Background and Aims*. There are limited data on the differences in diagnostic yield between 25-gauge and 22-gauge EUS-FNA needles. This prospective study compared the difference in diagnostic yield between a 22-gauge and a 25-gauge needle when performing EUS-FNA. *Methods*. Forty-three patients with intraluminal or extraluminal mass lesions and/or lymphadenopathy were enrolled prospectively. EUS-FNA was performed for each mass lesion using both 25- and 22-gauge needles. The differences in accuracy rate, scoring of needle visibility, ease of puncture and quantity of obtained specimen were evaluated. *Results*. The overall accuracy of 22- and 25-gauge needle was similar at 81% and 76% respectively (N.S). Likewise the visibility scores of both needles were also similar. Overall the quantity of specimen obtained higher with the 22-gauge needle (score: 1.64 vs. *P* < .001). However the 25-gauge needle was significantly superior to the 22-gauge needle in terms of ease of puncture (score: 1.9 vs. 1.29, *P* < .001) and in the quantity of specimen in the context of pancreatic mass EUS-FNA (score: 1.8 vs. 1.58, *P* < .05). *Conclusion*. The 22-gauge and 25-gauge needles have similar overall diagnostic yield. The 25-gauge needle appeared superior in the subset of patients with hard lesions and pancreatic masses.

## 1. Introduction

Endoscopic ultrasound-guided fine needle aspiration (EUS-FNA) has become well established as a technique for sampling lesions within the gastrointestinal tract and adjacent organ including pancreatic tumors, abdominal lymph nodes, adrenal tumors, mediastinal masses, and gastrointestinal submucosal tumors. EUS-FNA provides cytological or histological diagnosis for such lesions and has high diagnostic accuracy, sensitivity, and specificity of 80–90%, 90%, and 100%, respectively [1–4]. 

 The diagnostic accuracy of EUS-FNA is influenced by several factors, such as anatomical location and stiffness of the target lesion, the experience of the endoscopist, and the angle of the tip of echoendoscope [5–9]. To overcome these limitations and obtain adequate tissue sampling, a variety of EUS-FNA needle devices have been developed, and 19-, 22-, and 25-gauge configurations are available. The 22-gauge and 19-gauge needles are commonly used for EUS-FNA and have demonstrated high diagnostic accuracy rates. There are limited comparative studies on the clinical impact of different needle sizes [8–10]. It remains unclear whether a specific needle size will be more suitable for a particular type of lesion. 

 The aim of this nonrandomized prospective cohort study was to compare the 22-gauge and 25-gauge needles in terms of differences in accuracy rate, needle visibility, ease of puncture, and quantity of the specimen obtained.

## 2. Patients and Methods

### 2.1. Patients

The study included prospectively recruited patients with unknown intra- or extraluminal mass lesions who were referred for EUS-FNA sampling between January 2005 and March 2008 at the Department of Endoscopy, The Jikei University School of Medicine, Tokyo, Japan. Patients with suspected diagnosis of lymphoma or autoimmune pancreatitis in which abundant samples were required for definitive diagnosis were excluded from this study, and EUS-guided Trucut biopsy was instead performed for these lesions in our institution. The study was approved by the institution ethics committee and all patients gave written informed consent. This was a nonrandomized cohort study.

### 2.2. Method of Tissue Sampling with EUS-FNA

EUS was performed using the curvilinear echoendoscope (GF-UC2000P or GU-UCT240-AL5; Olympus Medical Systems, Tokyo, Japan) under conscious sedation using intravenous midazolam and pethidine. All EUS-FNA procedures were digitally videotaped to allow subsequent blinded evaluation of the needle visibility and the ease of puncture. After the lesion was carefully inspected and vessel interposition along the puncture route excluded by colour Doppler ultrasound, EUS-FNA was performed twice using the 22-gauge needle and 25-gauge needle (Echotip; Wilson-Cook, Winston-Salem, NC, USA) by a single experienced endosonographer (H.I). The order of needles used for EUS-FNA for all patients in our study is 22 G needle followed by 25 G. During each puncture, the needle traversed the lesion to and fro 10 times with negative suction applied using a 10 mL syringe. The aspirated specimen was then placed on glass slides and fixed in absolute ethanol solution for cytological Papanicolaou staining in all cases except for patients with submucosal lesions that were suspected to be gastrointestinal submucosal tumour (GIST). For cases of suspected GIST, the aspirated material was placed into formalin solution for histological examination since histology with immunohistochemistry was necessary to establish c-kit (CD-117), CD-34, actin, desmin, and S-100 positivity. The specimens obtained from the first and second punctures were labeled according to the aspiration sequence and sent to experienced cytopathologists who were blinded to type of the needles used. The adequacy of the specimen obtained was judged by the presence of macroscopic material without the presence of a cytopathologist. After the second aspiration, if further aspiration was needed to ensure adequacy of specimens, the 22-gauge needle was used and EUS-FNA was repeated until adequate specimen was obtained. The results of the first and second aspirations were compared.

### 2.3. Evaluation of EUS-FNA Puncture and Specimen Adequacy (Table 1)

(i) Needle Visibility and Ease of Puncture

The digitally recorded EUS-FNA procedures were reviewed by another experienced endosonographer (Y.U) who was blinded to the type of needle used. The characteristics of visibility of the needle and ease of puncture were documented for the first and second punctures with the two different needles. These characteristics were scored qualitatively as poor (scored 1), good (scored 2), and excellent (scored 3).

(ii) Adequacy of EUS-FNA Specimens

The characteristics of quantity of obtained specimens were reported by cytopathologists who were blinded to the type of needle used. The characteristics were scored qualitatively as poor (scored 1), good (scored 2), and excellent (scored 3).

 The final diagnosis was based on the results of EUS-FNA and surgery. The accuracy and each score were evaluated and compared between the 22-gauge and 25-gauge needles.

## 3. Statistics

For statistical analysis, the McNemar test was used for pairwise comparison of accuracy of EUS-FNA using 22-gauge needle and 25-gauge needle, and Wilcoxon signed rank test was used for pair-wise comparison of each score. For all test, a *P*-value less than .05 was regarded as statistically significant (using Stata version 10 software, StataCorp, Texas, USA).

## 4. Result

A total of 43 patients were recruited. The final diagnoses were gastric GIST in 12 patients, gastric leiomyoma in 7, gastric aberrant pancreas in 1, intra-abdominal schwannoma in 3, pancreatic cancer in 6, chronic pancreatitis in 6, malignant lymphadenopathy in 3, benign lymphadenopathy in 3, lung cancer in 1, and other in 1. Final diagnoses were obtained by surgery in the all patients with GIST and three with pancreatic cancer. In the remaining patients, the final diagnoses were based on the results of EUS-FNA, including additional EUS-FNA. The mean diameter of mass lesions in all patients was 25.7 mm in the long axis. The mean diameters of submucosal tumor and pancreatic lesions were 25.7 mm and 22.4 mm in their long axis, respectively (Table 2). 

Adequate material for cytological or histological evaluation was obtained in 35 lesions using 22-gauge needles, and 33 with 25-gauge needles. As a result, the overall diagnostic accuracy of the 22-gauge needle and the 25-gauge needle was 81.4% (35/43) and 76.7% (33/43), respectively, (N.S). In 43 patients, the mean score of visibility of the 22- and 25-gauge needles was 1.74 and 1.76 (N.S). However, the mean score of ease of puncture using the 25-gauge needle was significantly higher than that of the 22-gauge needle (1.9 versus 1.29, *P* < .001). On the other hand, overall the score of quantity of specimen using the 25-gauge needle was significantly lower than the 22-gauge needle (1.5 versus 1.64, *P* < .001) (Table 3). 

In twenty cases of gastric submucosal tumor, GISTs were diagnosed in 12, leiomyoma in 7, and aberrant pancreas in 1. The specimens were obtained using 22- and 25-gauge needles and evaluated by histological examination with immunohistochemistry to establish c-kit (CD-117), CD-34, actin, desmin, and S-100 positivity. Adequate materials for immunohistochemical evaluation were obtained in 16 out of 20 gastric submucosal tumors with 22-gauge needles and in 12 with 25-gauge needles. As a result, diagnostic accuracy for submucosal tumor with 22-gauge needle was higher than 25-gauge needle (80% (16/20) versus 60% (12/20)), although the difference was not statistically significant. The mean score of quantity of obtained specimen using the 22-gauge needle for submucosal tumor was significantly higher than 25-gauge needle (1.7 versus 1.3, *P* < .001). All punctures using the 22- and 25-gauge needles were successfully performed in all cases of submucosal tumor. However, regarding ease of puncture, the mean score of the 25-gauge needle was significantly higher than 22-gauge needle (1.95 versus 1.3, *P* < .001). The reviewers noted that when 22-gauge needle was used for submucosal tumor, the targeted lesion and gastric wall were more likely to move together with the needle, although it did not easily bent during the procedure. There was no significant difference for the score of visibility of needle between 22- and 25-gauge needles (Table 4).

**Figure 1:**
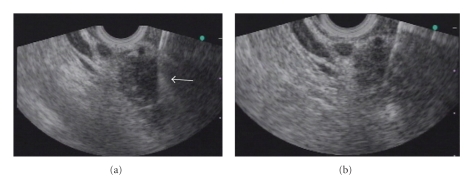
EUS-FNA images for pancreatic head cancer using (a) 22-gauge and (b) 25-gauge needles. The tip of the 22-gauge needle was bent within the lesion during the puncture, and no material was obtained. The arrow indicates the bent tip of the 22-gauge needle. The 25-gauge needle tip remained straight during the EUS-FNA procedure (arrow), and pancreatic adenocarcinoma was diagnosed with EUS-FNA using the 25-gauge needle.

 Six pancreatic cancers and 6 cases of chronic pancreatitis were diagnosed from 12 patients with pancreatic mass lesions. The location of pancreatic mass lesions was head of pancreas in 8, body in 3, and tail in 1. Adequate material for cytological evaluation was obtained in 9 cases with 22-gauge needle and 11 with 25-gauge needles. The diagnostic accuracy for pancreatic mass with 25-gauge needle was relatively high as compared with 22-gauge needle (91.5% (11/12) versus 75% (9/12)), although the difference was not statistically significant. Although the 22-gauge needle could not obtain material in one case of pancreatic cancer and one case of chronic pancreatitis, the 25-gauge needle could obtain adequate material for cytological diagnosis in these two cases. A representative case was shown in Figure 1. In addition, the 25-gauge needle could acquire adequate material in a case of chronic pancreatitis; whereas 22-gauge needle could not provide definitive diagnosis because of inadequate small materials. The opposite result was noted in another case of chronic pancreatitis. Finally, 22-gauge needle could obtain no material in 2 (16.7%) cases of pancreatic lesions and obtain inadequate materials for definitive diagnosis in 1 (8.3%). On the other hand, 25-gauge needle could obtain material in all cases of pancreatic lesions, and it could not obtain adequate materials for definitive diagnosis in only 1 case (8.3%). Therefore, the mean score of quantity of obtained specimens using 25-gauge needle for pancreatic mass lesions was significantly higher than 22-gauge needle (1.58 versus 1.83, *P* < .05). In addition, regarding ease of puncture, the mean score of 25-gauge needle was significantly higher than the 22-gauge needle (1.83 versus 1.17, *P* < .001). The reviewer noted that when 25-gauge needle was used for pancreatic mass lesion, the needle tended to penetrate the lesion more perpendicularly without bending, and the lesion was not more likely to be pushed away from transducer of echoendoscope. On the other hand, 22-gauge needle tended to bend during the procedure. EUS-FNA using 22-gauge was not successful in one case of chronic pancreatitis because of the firmness and rigidity of the lesion, although successful puncture using the 25-gauge needle was achieved in all 12 cases with pancreatic mass. There was no significant difference for the score of visibility of needle between 22- and 25-gauge needles (Table 5) 

There were no procedure-related complications irrespective of the type of needles used.

## 5. Discussion

EUS-FNA has high and well-established diagnostic accuracy with a very high safety [11–14]. However, its diagnostic accuracy might be limited by several factors including anatomical location and consistency of lesions. 

 When a hard tumor is punctured, bending of the needle may occur resulting in the needle tip not being visualized on the ultrasonic image [15]. In addition, the needle tip will also not be visualized if the tip of the echoendoscope cannot be securely fixed in the duodenum and is pushed away from the targeted lesion during EUS-FNA. These limitations could lead to inadequate sampling with EUS-FNA [9, 15]. The diagnostic yield of EUS-FNA for pancreatic head masses is lower than that of mediastinal lesions [16–19]. This may be explained by the fact the for pancreas head masses, EUS-FNA is performed from the duodenum, where the tip of the echoendoscope may be bent and hence less stable. Gastric submucosal masses including GIST are also difficult to puncture because the gastric wall tends to move considerably together with the needle. Furthermore abundant tissue is necessary in the diagnosis of submucosal tumours because immunohistochemical staining is required to differentiate GIST from leiomyoma and neurogenic tumor. This difficulty in EUS-FNA and the need to acquire abundant tissue could also result in a lower diagnostic accuracy rate in submucosal tumors [15, 20]. 

 In order to overcome these limitations, a variety of EUS-FNA needle devices have been developed and are now available in 19-, 22-, and 25-gauge sizes. EUS-FNA using the 22-gauge needle has been the most extensively studied and shown to have a high diagnostic accuracy rate of 95 to 100% for pancreatic cancer [18, 19] and 82 to 91% for GIST, respectively [20, 21]. However, there are only few reports on the clinical impact of EUS-FNA using the 25-gauge needle [22]. Furthermore, there are very limited comparative studies to clarify which needle size is more suitable for a particular type of lesion [8–10, 22]. Therefore, we embarked on a comparative study on the use of 25- and 22-gauge needles for EUS-FNA. 

 Our results showed that the overall diagnostic accuracy of the 22-gauge needle was similar to the 25-gauge needle (81.4% versus 76.7%, *P* > .05). However the score of quantity of specimen obtained using the 22-gauge needle was significantly higher than the 25-gauge needle (1.64 versus 1.5). These results suggest that a larger diameter of needle has the advantage of acquiring more tissue, as compared with a smaller diameter needle. Indeed, this advantage became more evident in patients with submucosal tumor, where abundant material was necessary for diagnosis by histology with immunohistochemistry. However, the score of ease of puncture by the 25-gauge needle was significantly higher than 22-gauge needle (1.9 versus 1.29). This statistically significant difference was also seen in patients with submucosal tumor (1.3 versus 1.95). Nonetheless, diagnostic yield of EUS-FNA using 22-gauge needle was superior to 25-gauge needle in patients with submucosal tumor. The ease of puncture using the 25-gauge needle might not influence the accuracy and the quantity of obtained specimen in patients with submucosal tumors. 

 Diverging results were seen in patients with pancreatic masses. The diagnostic accuracy of the 25-gauge needle for a pancreatic mass lesion was significantly higher than that of the 22-gauge needle (91.7% versus 75%). The score of quantity of obtained specimen obtained using the 25-gauge needle was also significantly higher than the 22-gauge needle. The score of ease of puncture using the 25-gauge needle was significantly higher than 22-gauge needle. In patients with pancreatic mass, a larger diameter of needle might not necessarily lead to better results. The ease of puncture using the 25-gauge needle in pancreatic masses, which tend to be hard in consistency, may account for the increased diagnostic accuracy and quantity of obtained specimen in pancreatic lesions. 

 There were clearly different outcomes in the diagnostic yield between the different needle types for submucosal tumors and pancreatic lesions. Possible reasons for the different outcomes might be related to the characteristics of difficulty of puncture for each particular lesion. The needle and gastric wall were likely to move together during puncture when 22-gauge needle was used. As a result, several puncture attempts were needed before the needle could traverse deep into the lesion. However, 22-gauge needle could move without bending once it had successfully penetrated into the lesion. Because all punctures using 22- and 25-gauge needles were performed successfully for submucosal tumors in this study, the ease of puncture using the 25-gauge needle might not influence the diagnostic accuracy and quantity of the specimen obtained. Pancreatic tumors are known to be extremely firm, which may not only resist needle penetration but also prevent adequate tissue sampling. The bent shaft of the echoendoscope when it rested on the duodenal wall during EUS-FNA for pancreatic head mass could prevent smooth movement of the needle within the accessory channel [5, 9, 23]. Itoi et al. have shown that the diagnostic accuracy of the 22-gauge needle for pancreatic mass in the head and uncinate process was higher than that of the 19-gauge needle [8]. Recently, Sakamoto et al. also showed that the 25-gauge needle was superior to the 22-gauge needle for overall diagnostic accuracy in the context of EUS-FNA of pancreatic head and uncinate lesions [22]. In our 8 out of 12 pancreatic head lesions, 25-gauge needles tended to penetrate and move more perpendicular into pancreatic masses without bending as compared with 22-gauge needles. In addition, 22-gauge needle could not penetrate into the pancreas in patients with chronic pancreatitis because of its hardness. Thus, smaller diameter needles, which are sharper, might have an advantage over larger ones in EUS-FNA for pancreatic mass. Therefore, the ease of puncture using 25-gauge needle might influence the diagnostic accuracy and quantity of obtained specimen from pancreatic lesions. 

 In conclusion, no significant differences were detected in the diagnostic accuracy and visibility between 22- and 25-gauge needles. However, ease of puncture using 25-gauge needle was significantly superior to 22-gauge needle. The quantity of the specimen obtained was better with the 25-gauge needle for pancreatic lesions; whereas the 22-gauge needle was better for submucosal tumors. The decision to use either the 22-gauge or 25-gauge needle should be based on the characteristics of the targeted lesions. However, our study has several limitations, including small number of patients, lesions in various locations, and different types of pathology. Therefore, further studies are warranted to clarify the feasibility of different types of EUS-FNA needles for different lesions.

## Figures and Tables

**Table 1:** Scoring of visibility of needle, ease of puncture, and quantity of specimen obtained.

Visibility of needle	0: poor	Needle tip is absolutely invisible.
	1: good	The needle tip is visible, although invisible intermittently during the procedure.
	2: excellent	The needle tip is visible throughout the procedure.

Ease of puncture	0: poor	Impossible to puncture.
	1: good	The needle bends during the procedure, or the target lesion is pushed away from the transducer of the echoendoscope at the time of puncture.
	2: excellent	The puncture is done effortlessly and the needle never bends during procedure.

Quantity of the specimen obtained	0: poor	No specimen
	1: good	Although a small specimen is obtained, definitive diagnosis is difficult.
	2: excellent	Adequate for definitive diagnosis.

**Table 2:** Final diagnosis.

Diseases	*n*
Gastric GIST	12
Gastric leiomyoma	7
Gastric aberrant pancreas	1
Pancreatic cancer	6
Chronic pancreatitis	6
Intra-abdominal schwanaoma	3
Malignant lymphadenopathy	3
Benign lymphadenopathy	3
Lung cancer	1
Others	1

**Table 3:** Comparison of EUS-FNA with the 22-gauge and 25-gauge needles.

	22G	25G	*P*
Diagnostic accuracy	81.4% (35/43)	76.7% (33/43)	N.S
Visibility of the needle	1.74 ± 0.44	1.76 ± 0.43	N.S
Ease of puncture	1.29 ± 0.55	1.9 ± 0.29	*P* < .001
Quantity of the specimens obtained	1.64 ± 0.68	1.5 ± 0.73	*P* < .001

**Table 4:** Comparison of EUS-FNA of submucosal tumours with the 22-gauge and 25-gauge needles.

	22G	25G	*P*
Accuracy	80% (16/20)	60% (12/20)	N.S
Visibility of the needle	1.7 ± 0.45	1.65 ± 0.48	N.S
Ease of puncture	1.3 ± 0.46	1.95 ± 0.22	*P* < .001
Quantity of the specimens obtained	1.7 ± 0.64	1.3 ± 0.84	*P* < .001

**Table 5:** Comparison of EUS-FNA of pancreatic masses with the 22-gauge and 25-gauge needles.

	22G	25G	*P*
Accuracy	75% (9/12)	91.7% (11/12)	N.S
Visibility of the needle	1.83 ± 0.37	1.83 ± 0.37	N.S
Ease of puncture	1.17 ± 0.55	1.83 ± 0.37	*P* < .001
Quantity of the specimens obtained	1.58 ± 0.76	1.83 ± 0.37	*P* < .05
